# COVID-19 contact tracing and quarantine policies in the Indo-Pacific Region: A mixed-methods study of experiences of public health professionals

**DOI:** 10.1371/journal.pgph.0003121

**Published:** 2024-05-31

**Authors:** Md. Saiful Islam, Florian Vogt, Catherine King, Meru Sheel

**Affiliations:** 1 National Centre for Epidemiology and Population Health, ANU College of Health and Medicine, The Australian National University, Canberra, Australian Capital Territory, Australia; 2 School of Population Health, Faculty of Medicine and Health, University of New South Wales, Sydney, Australia; 3 Sydney School of Public Health, Faculty of Medicine and Health, The University of Sydney, Sydney, New South Wales, Australia; 4 Sydney Infectious Diseases Institute, Faculty of Medicine and Health, The University of Sydney, Sydney, New South Wales, Australia; University of Embu, KENYA

## Abstract

Contact tracing and quarantine are valuable public health tools to prevent the transmission of SARS-CoV-2 and control the epidemic. Many low-and middle-income countries (LMICs) adopted global contact tracing and quarantine guidelines but were unable to contextualise the guidance into policies and practices that were relevant to their setting. Therefore, we examine contact tracing policies and practices in the Indo-Pacific region and the need to design context-specific policies. We conducted a mixed-methods study, including a cross-sectional online survey followed by key-informant interviews (KIIs). Using convenience snowball sampling, we invited public health professionals primarily involved in COVID-19 pandemic response from the Indo-Pacific region. We undertook descriptive analyses using counts and percentages for survey data and framework analysis for qualitative data. Seventy-seven public health professionals participated in the survey, of whom ten also participated in the KIIs. The study identified significant gaps between policies and the local contexts. Factors that broaden the gaps were limited knowledge of the changing dynamics of COVID-19 transmission, poor leadership, and coordination, little or no formal training on contact tracing, poor understanding of the guideline recommendations, limited resources, community resistance and mistrust, social stigmatisation and fear of being ostracised, and discrimination. This study revealed substantial disparities between policies and local contexts, significantly influencing policy implementation at national, provincial, and district levels across the studied countries. To bridge these gaps, we advocate for national contact tracing and quarantine guidelines explicitly addressing the quarantine needs of specific demographics, including children, pregnant women, prisoners, and individuals affected by social exclusion issues. Furthermore, we propose strengthening contact tracing training programs, urging revised guidelines to account for social, cultural, and infrastructural nuances influencing contact tracing and quarantine implementation. We also recommend engaging local NGOs, faith-based organisations, and local administrations to reinforce community connections and strengthen contact tracing.

## Introduction

COVID-19 is an emerging infectious disease caused by SARS-CoV-2 that transmits from person-to-person through respiratory droplets and aerosols [1]. Contact tracing and quarantine are essential public health tools to prevent transmission of infectious diseases and control outbreaks. Contact tracing involves identifying, assessing, and managing contacts defined as people who have exposure to someone infected with an infectious disease, and quarantine involves the separation of contacts [2]. Quarantine measures can be voluntary or legally enforced by law and often applied to an individual, family, group, or community level [3].

During the initial phases of the COVID-19 pandemic, contact tracing and quarantine played pivotal roles in controlling SARS-CoV-2 morbidity and mortality [4, 5]. In a natural experimental study examining the effectiveness of COVID-19 contact tracing and quarantine in England during 2020, Fetzer and Graeber demonstrated that timely and comprehensive contact tracing could potentially reduce approximately 63% of new infections and 66% of COVID-19 related mortality [6]. A systematic review corroborated these findings, indicating that contact tracing and quarantine prevented 44% to 81% of new cases and reduced mortality by 31% to 63% [7]. A more recent mathematical model by Wu et al. (2023) further supported these outcomes, suggesting that implementing contact tracing and quarantine policies can reduce the overall size of the epidemic [8]. Additionally, in a recent systematic review assessing the real-world effectiveness of these measures, consistent findings emerged across studies, indicating that testing, contact tracing, and quarantine measures were associated with decreased morbidity and mortality. The effectiveness was observed through reduced case growth, lower cases per capita, and decreased population-level mortality rates [9].

Despite the known effectiveness of contact tracing, it remains a resource-intensive process, requiring a skilled workforce and good public health infrastructure. Likewise, quarantine requires space for separation, food and medicine supplies, effective communication, and a trained workforce to manage them. Therefore, the implementation of contact tracing and quarantine can put significant pressure on health systems, especially in low-and middle-income countries (LMICs) or resource-constrained settings [10].

The implementation of global and national contact tracing and quarantine policies can vary significantly at the sub-national level, especially in decentralised health systems where infrastructural capabilities differ [11]. These challenges were magnified during the COVID-19 pandemic, wherein many LMICs adopted or attempted to adopt global policies, guidance and norms, developed by global agencies, for example, the World Health Organization (WHO), and the United States Centers for Disease Control and Prevention (CDC). However, our collective field experiences suggested that numerous LMICs encountered challenges contextualising this guidance into policies and practices relevant to their unique circumstances [10, 12]. There was an expectation to align policies with those from resource-rich settings or past experiences with other infectious diseases. Many countries encountered difficulties sustaining contact tracing efforts as the COVID-19 caseload surged [12, 13].

Numerous studies have investigated community perceptions and experiences related to isolation, quarantine, and the experiences of public health responders, utilising qualitative research methods within specific country contexts [12, 14–16]. However, at the time this study was initiated, there were no studies examining contact tracing and quarantine policies from the perspective of public health professionals. We used a mixed-methods study to collect data on contact tracing and quarantine policies in the Indo-Pacific region and collect data from LMICs in the region. This study aimed to explore future opportunities for designing and implementing contact tracing policies, offering perspectives from public health professionals, epidemiologists, and frontline workers directly involved in these efforts. Our study aimed to examine contact tracing policies and practices in the Indo-Pacific region, to understand the extent to which context-specific policies were being used and explore any related barriers.

## Methodology

### Study design, study setting and population

**Fig 1:**
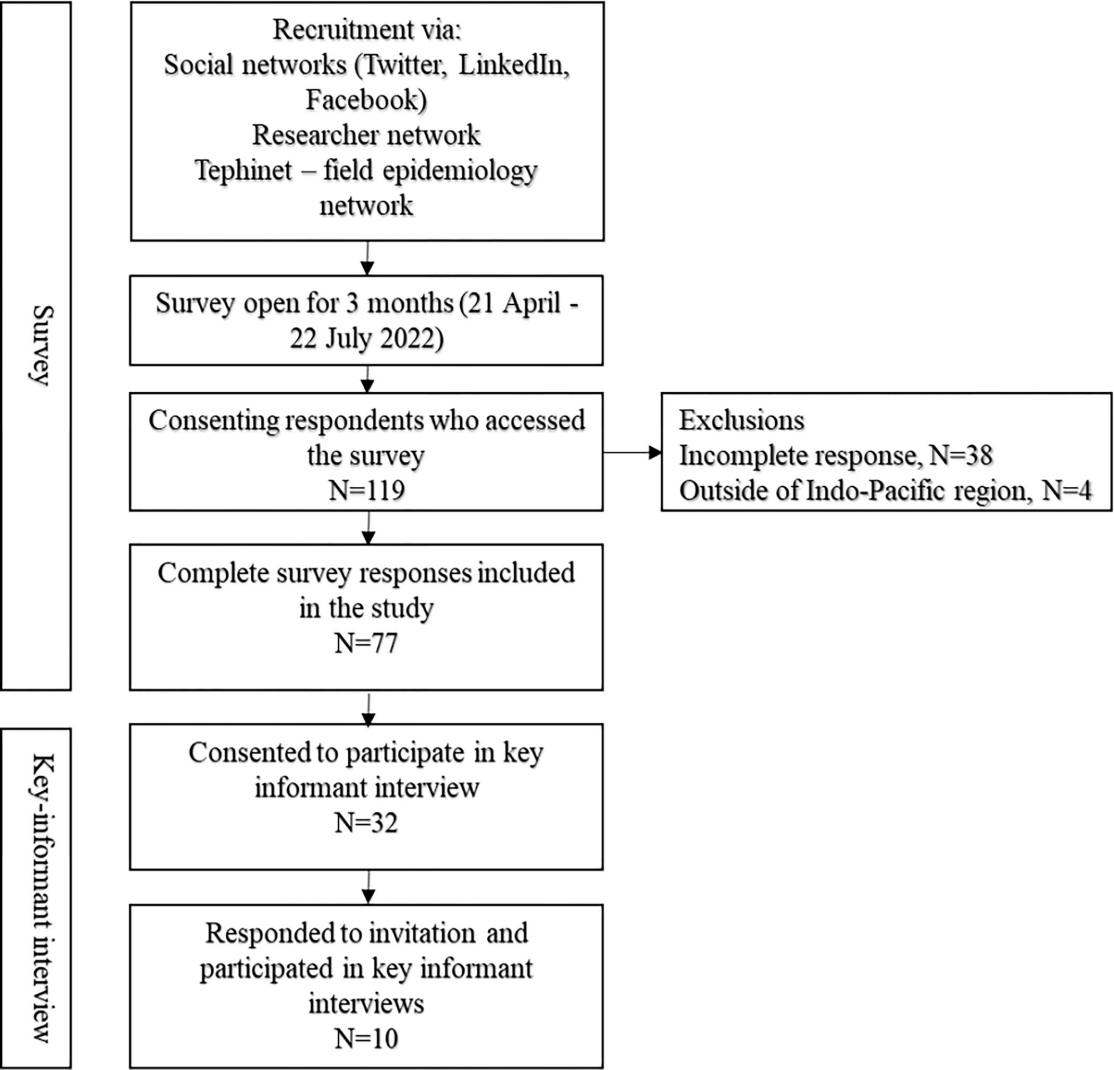
Study flow chart.

The study, conducted between March and October 2022, employed a mixed-methods approach, commencing with a cross-sectional online survey and followed by a qualitative study utilizing key informant interviews (KIIs) of survey participants (Fig 1). We invited professionals aged 18 years and above who were involved in COVID-19 contact tracing and quarantine activities. The target population was professionals working within Ministries of Health, international organisations’ national representatives, research organisations, and local health service workers from the Indo-Pacific region, including countries such as Australia, Bangladesh, Cambodia, Fiji, India, Indonesia, Japan, Kiribati, Malaysia, Nepal, New Zealand, Palau, Papua New Guinea, the Philippines, Thailand, and Vietnam. We limited the scope of the study to these countries due to cultural, geographical, and economic connection and similarities, and due to a similar approach to COVID-19 mitigation measures in 2020 [17]. Recruitment for the online survey was done by sharing the link to the survey via investigators’ networks, social media, and operational partners-TEPHINET (Training Programs in Epidemiology and Public Health Interventions Network, *(*https://www.tephinet.org/*)*- a global professional network of field epidemiology training programs. Recruitment was continued through convenience sampling, including snowballing, as the link to the survey was shared across professional networks and among collaborators.

### Data collection

The quantitative online survey link was open for 3 months (21 April-22 July 2022) and included both structured and unstructured questions. The survey contained four key domains: (a) socio-demographic characteristics and professional experience; (b) COVID-19 contact tracing and quarantine policies and practices; (c) contact tracing processes; (d) implementation of quarantine policies and the challenges experienced. Feedback on the questionnaire was sought from the study team and TEPHINET. The questionnaire was piloted and revised before implementation. Data were collected and collated online using REDCap, a web-based application for public health research, databases and projects [18].

At the end of the survey, respondents were given an option to participate in a KII. Those who responded ‘Yes’, were invited to participate in a short interview via email. We sought informed oral consent before the interview. We used an open-ended topic guide to support the interview which was developed by a social scientist and epidemiologists and piloted in an LMIC for cognitive understanding. The topic guide included participants’ current role in contact tracing, different steps of contact tracing and identification of cases and contacts, attitudes toward contact tracing policies, gaps, and challenges in contact tracing. Interviews were conducted by MSI using video conferencing software and recorded for note taking purposes. MSI was aware of each country’s context and health infrastructure, which allowed him to practice cultural and social considerations during the data collection process.

### Data management and analysis

**Fig 2:**
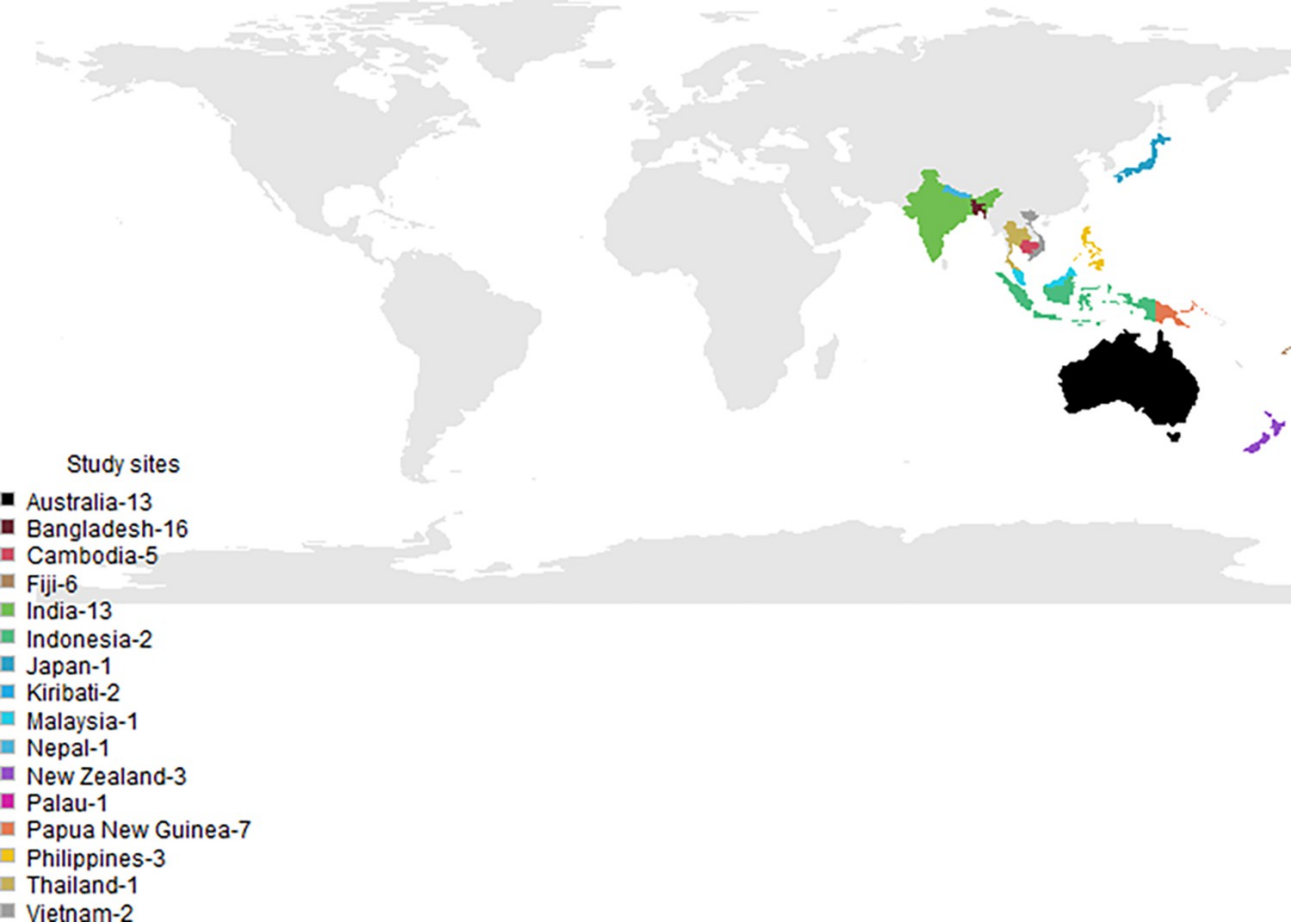
Study sites for COVID-19 contact tracing and quarantine policies in the Indo-Pacific Region, 2022. The base map was created with R package “rworldmap” (https://cran.r-project.org/web/packages/rworldmap/). Country borders are derived from natural Earth data v 1.4.0 in “rworldmap”.

We conducted descriptive analyses on the survey data, utilising counts and percentages. For analytical purposes, we categorised the respondents’ countries into ’LMIC’ and ’high-income’ groups, following the World Bank’s gross national income (GNI) criteria. LMICs encompass countries with a GNI per capita between $1,136 and $13,845, while high-income economies are those with a GNI per capita of $13,846 or more [19]. Chi-square test and Fisher’s exact test was used to compare if the differences between LMICs and high-income countries were statistically significant. Survey data were analysed using STATA v.13 and the open-source statistical package R version 4.2.0. We used the R package ‘rworldmap’ to develop a map (Fig 2).

For qualitative data, we used both deductive and inductive coding for the analysis; the former was guided by the question categories and the latter emerged from the KIIs findings [20]. MSI transcribed the interview audio recordings verbatim before deleting, organised the notes, and compiled them into a written report format. MSI and CK independently read all the interview transcriptions and reviewed one transcript in detail and developed an initial code list. MSI coded the rest of the interviews using the code list as an initial basis. MSI created new codes to accommodate new findings that were beyond the scope of the initial codes. These were discussed with CK, MS and consensus reached on their final inclusion. This iterative process minimised individual biases and enhanced the reliability of our qualitative analysis.

We used a framework approach, which is well suited to policy description and analysis to structure and further analyse data in Microsoft Excel [21]. MSI entered the interview IDs into rows, the codes in the column headings and then extracted data under the relevant codes. The output of this matrix spreadsheet allowed us to summarise data systematically and analyse according to sources, codes and by themes [22]. This approach also allowed us to compare data by pre-produced codes and emerging themes and sub-themes across countries. All authors reviewed the categories and sub-categories for consensus and reliability.

### Ethics approval and consent to participate

Participation in this study was voluntary. Respondents were directed to plain language information sheets available online in English before accessing the survey. Interested participants were directed to complete the informed consent form via Research Electronic Data Capture (REDCap). Consenting participants were then directed to access the survey. Only those who provided consent were able to access and submit the survey. At the end of the survey, participants were asked if they would be interested and willing to participate in a KII. For those consenting participants, another participant information sheet and an oral consent form were shared via email. Then at the time of the online interview, the information sheet was again read out to participants to ensure they understood the study. Thereafter participants expressed their oral consent to participate in the KII. Ethical approval to conduct the study was approved by the Australian National University Human Research Ethics Committee (protocol number 2021–795)

## Results

### Survey results

One hundred nineteen public health professionals consented to participate in the survey. Of those, data from 77 (65%) respondents was included in the final analyses. Of the 42 surveys that were excluded, 90.5% (38) had considerable missing data, or respondents (N = 4, 9.5%) were outside of the Indo-Pacific region and did not meet the study eligibility criteria. Of the 77 respondents, 49% (38/77) were male and the median age of all respondents was 36 years (range 22–65 years) (Table 1). Data from respondents represents 16 countries (Fig 2), of which the majority were from Bangladesh (21%;16/77), India (17%; 13/77) and Australia (17%;13/77). Seventy-eight percent (60/77) of respondents were from LMICs. Sixty-nine percent (53/77) of the respondents worked within a Ministry of Health (MoH) or another government agency. Thirty-six percent (19/53) of the government employees were from the national level, 34% (18/53) were divisional/provincial, and 30% (16/53) were from district and sub-district levels (Table 1).

**Table 1:** Socio-demographic characteristics and contact tracing experience of survey respondents, N = 77, 2022.

Characteristic	Respondents n (%)
**Gender of respondent**	
Male	38 (49)
Female	38 (49)
Prefer not to say	1 (2)
**Income groups**	
Low and middle-income countries	60 (78)
High-income countries	17 (22)
**Age of respondent (years)**	
20–29	16 (21)
30–39	35 (45)
40–49	19 (25)
50 and above	7 (9)
**Type of organisation**	
Government organisation	53 (69)
Non-government organisation	10 (13)
WHO	2 (3)
UNICEF	1 (1)
Other	6 (8)
No response	5 (6)
**Work level in the government organisation**	
National	19 (36)
Divisional/State/Province	18 (34)
District/Sub-district	16 (30)
**Role in the current organisation** *	
Epidemiologist	28 (36)
Contact Tracing Officer	28 (36)
Public Health Officer	20 (26)
FETP Fellow/alumni	24 (31)
Nurse/medical doctor/intern	21 (27)
Community health worker	2 (3)
Volunteer	4 (5)
Other	15 (19)
**Year of experience as a COVID-19 contact tracer**	
<1 year	21 (27)
1>2 years	38 (49)
> years	18 (24)
**Had contact tracing experience before COVID-19**	
Yes	39 (51)
No/unsure	38 (49)
**Total years of contact tracing experience**	
>2 years	12 (16)
2>3 years	11 (14)
3 years and above	15 (19)
No response or no experience	39 (51)
**Received formal training for contact tracing and quarantine**	
Yes	38 (49)
No	36 (47)
Unsure	2 (3)
No response	1 (1)
**Usefulness of the training**	
Useful	37 (97)
Not useful	1 (3)
**Would formal training for contact tracing and/or quarantine have been useful in current role**	
Yes	30 (39)
No	6 (8)
No response	41 (53)
**Preferred mode for future training**	
Face to face	6 (8)
Simulation	7 (9)
Online	8 (10)
Face-to-face and/or simulation or online	7 (9)
No response	49 (64)

*People worked in multiple roles. Multiple answers were accepted and therefore, the total number and the % total more than 100.

### Current role, professional experience, and training

**Fig 3:**
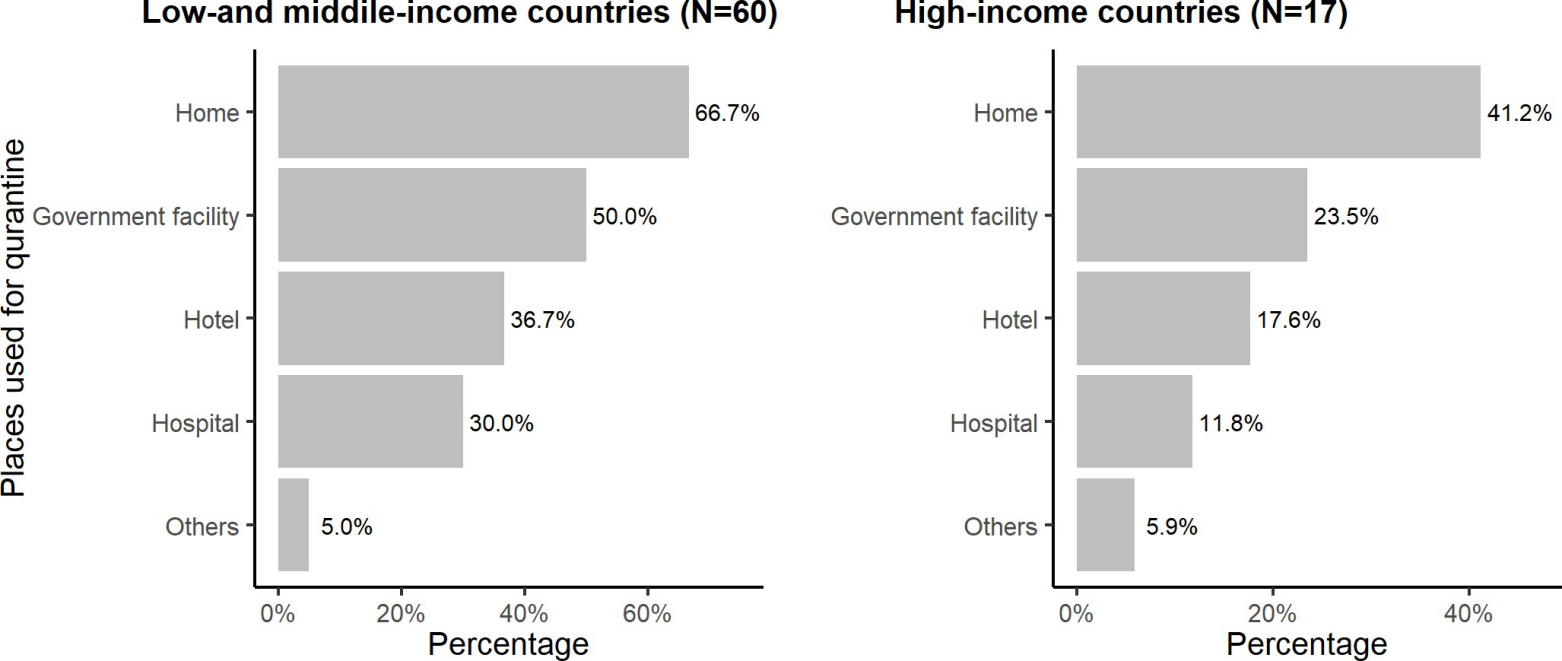
Places used for quarantine in low-and middle-income countries vs high income countries, 2022.

**Fig 4:**
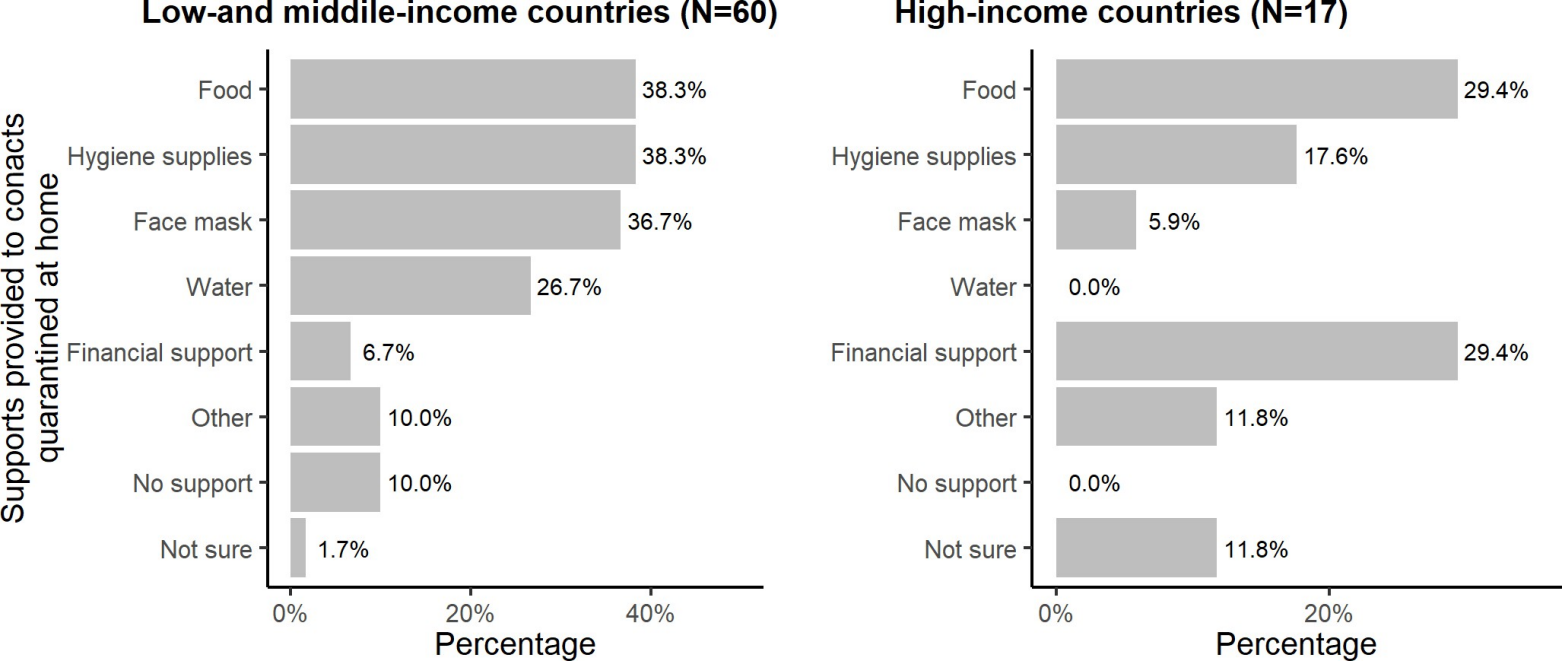
Supports provided to contacts quarantined at home in low-and middle-income countries vs high-income countries, 2022.

During the survey, some of the respondents identified as having more than one role. Among the respondents, 36% (28/77) self-identified as contact tracing officers, 36% (28/77) as epidemiologists, 26% (20/77) as public health officers, 31% (24/77) as Field Epidemiology Training Program (FETP) fellows or alumni, 27% (21/77) as nurses and doctors including interns, and 27% (21/77) as community health workers, volunteers, and others. As most respondents were public health professionals even prior to the pandemic, just over half (51%, 39/77) had prior contact tracing experience. Forty-nine per cent (38/77) of the respondents reported that they had received formal training for contact tracing, and among those who received the training, almost all (97%; 37/38) found it very useful (Table 1). In LMICs, more people were quarantined at home (67% vs 41%) and government facilities (50% vs 24%) in comparison to HICs (Fig 3). About 29% of the respondents from HICs mentioned that they provided financial support to people quarantined at home whereas only 7% from LMICs mentioned that. In addition, 10% respondents from LMICs reported that they did not provide any support to people who were quarantined at home (Fig 4).

More than half of the respondents (52%; 40/77) reported that they routinely conducted contact tracing during COVID-19 (Table 2). Over 50% of the respondents (40/77) said they faced challenges while conducting contact tracing activities (Table 2). Table 3 showed that the participants from HICs had more years of experience in contact tracing than participants from LMICs, and the difference was statistically significant (P>0.01). The results also showed that more respondents from HICs received formal training for contact tracing compared to respondents from LMICs, and the difference was statistically significant (p<0.01).

**Table 2:** Contact tracing and quarantine policies, practices, and the challenges reported by survey respondents, N = 77, 2022.

Factors	Respondents n (%)
**Have contact tracing and quarantine guidance/policy**	
Only for contact tracing	3 (4)
Only for quarantine	4 (5)
For both	53 (69)
For neither	3 (4)
Not sure	5 (6)
No response	9 (12)
**Have contact tracing and quarantine standard operating procedure**	
Only for contact tracing	7 (9)
Only for quarantine	4 (5)
For both	44 (57)
For neither	7 (9)
Not sure	6 (8)
No response	9 (12)
**Routinely conduct contact tracing**	
Yes	40 (52)
No	10 (13)
No response	27 (35)
**COVID-19 contact tracing processes and practices changed over time**	
Yes	40 (52)
No	12 (16)
No response	25 (32)
**Use digital tools for contact tracing**	
Yes	40 (52)
No	11 (14)
No response	26 (34)
**Type of digital tools used**	
Go.Data	12 (16)
REDcap	1 (1)
Open Data Kit	1 (1)
Locally developed application	10 (13)
Manual data collection/Excel	12 (16)
Other	4 (5)
No response	37 (48)
**Use contact tracing applications**	
Yes	27 (35)
No	26 (34)
No response	24 (31)
**Usefulness of contact tracing application**	
Not at all useful	4 (5)
Moderately useful	14 (18)
Very useful	8 (11)
No response	51 (66)
**Put someone in quarantine**	
Yes	44 (57)
No	5 (7)
No response	28 (36)
**Place of quarantine** ^**1**^	
Home	47 (61)
Government facility	34 (44)
Hotel	25 (32)
Hospital	20 (26)
Other	4 (5)
**Supports provided to people quarantined at home** *	
Food	28 (37)
Water	16 (21)
Hygiene supplies	26 (34)
Face mask	23 (30)
Financial support	9 (12)
Nothing	6 (8)
Not sure	3 (4)
Medication and other supports	8 (11)
**Have faced challenges while conducting contact tracing and quarantine**	
Yes	40 (52)
No	1 (1)
No comment/response	36 (47)

*Multiple answers accepted

^1^ Multiple answers accepted.

**Table 3:** Contact tracing and quarantine practices and challenges by income groups, 2022.

Factors	Low and middle-income countries (N = 60)	High-income countries (N = 17)	
	Respondents n (%)	Respondents n (%)	P-value^2^
**Total years of experience in contact tracing**			
Less than two years	12 (37)	0 (0)	**0.01**
Two to less than three years	11 (33)	0 (0)	
Three years and above	10 (30)	5 (100)	
**Have received formal training for contact tracing**			
Yes	25 (42)	13 (76)	
No/unsure	34 (58)	4 (24)	**0.01**
**Usefulness of training**			
Yes	28 (87)	2 (50)	0.12
No	4 (13)	2 (50)	
**Have contact tracing and quarantine guidance/policy**			
Contact tracing or quarantine	6 (11)	1 (7)	
For both	39 (74)	14 (93)	0.23
No/not sure	8 (15)	0 (0)	
**Have contact tracing and quarantine standard operating procedure**			
Contact tracing or quarantine	10 (19)	1 (6)	
For both	31 (60)	13 (81)	0.35
No/unsure	11 (21)	2 (13)	
**Routinely conduct contact tracing**			
Yes	35 (80)	5 (83)	1.0
No	9 (20)	1 (17)	
**Use of applications for contact tracing**			
Yes	21 (47)	6 (75)	0.14
No	24 (53)	2 (25)	
**Usefulness of contact tracing applications**			
Not at all useful	3 (15)	1 (17)	
Moderately useful	11 (55)	3 (50)	1.0
Very useful	6 (30)	3 (33)	
**Have faced challenges while conducting contact tracing and quarantine**			
Yes	36 (90)	4 (80)	
No/ No comment	4 (10)	1 (20)	0.46

^2^ Fisher exact test was used.

### Policies and procedures

Most respondents reported their organisation had COVID-19 contact tracing policies (69%, 53/77) and standard operating procedures (SOPs) (57%, 44/77). When asked to differentiate, 4% (3/77) respondents said only contact tracing policies and 5% (4/77) reported that they only had policies for quarantine in their country. Ten percent (8/77) reported that they had neither or were unsure if they had any. Similarly, only 9% (7/77) respondents reported having an SOPs for contact tracing, 5% (4/77) only for quarantine, and 17% (13/77) said that they had neither or were unsure if they had any (Table 2).

### Findings from key-informant interviews

Among the 77 included survey participants, 32 initially agreed to be contacted for KII. However, interviews could only be conducted with 10 participants from eight LMICs who responded positively when contacted between July to October 2022. Overall, the KIIs provided more in-depth insights about the respondents’ everyday activities and roles that included COVID-19 case and contact monitoring and surveillance, contact investigation and quarantine, coordination of emergency response and contact tracing team, and data management at the national, state/province and district levels. Further, the KII findings provided rich data about participants’ thoughts towards contact tracing policies and procedures, along with the challenges they faced while implementing them.

### Case confirmation, contact identification and quarantine

Almost all the key informants reported using the WHO definition to define a case, namely a person with positive laboratory or rapid antigen test results suggestive of COVID-19 infection. However, the contact tracing processes were not uniform across all countries and provinces and differed according to context and resources. Unlike a ‘case’, the definition of a ‘contact’ had been revised and updated over time and varied by country. For example, one participant mentioned that they defined contact as individuals who had exposure to a case two weeks from the onset of symptoms.

“*So*, *we always identify people in contact with a case in the last two weeks of symptom onset*. *The family members*, *the people the case met in shops*, *local markets*, *and wherever the case visited were identified as contacts*. *We collected the phone numbers of all contacts and called them for symptom screening*.*”* A Medical Officer

The key informants reported that once contacts were traced, they were quarantined and followed up for 28 to 21 days initially, then 14 days to seven to five days. A few countries also conducted case-based contact tracing as explained by one participant:

"*At one point*, *we had a case who travelled from XX Island to Brisbane to one of the outer islands in this country where at that time no COVID-19 case had been detected*. *In that instance*, *we thought there would be a public health benefit of identifying the source of a delta outbreak and potentially avoiding any onward transmission*. *We then conducted a contact investigation*.*”* A Public Health Physician.

### Evolution of quarantine facilities

From the survey, we learned the types of facilities used for quarantining (Fig 3). The interviews revealed how the use of these evolved overtime. All but one participant mentioned that at the beginning of the COVID-19 pandemic, contacts were quarantined in hotels and hospitals. Some participants added that as the number of cases increased, the government started acquiring different institutions, empty buildings, TB hospitals, camps, schools, colleges, party halls and community centres, and using them as quarantine facilities; however, the quarantine process was not uniform across all countries and changed over time. Some participants mentioned that home quarantine policies came after the second wave of COVID-19 when the government could not manage quarantine facilities for larger numbers of people. In one country, many community organisations reportedly came forward to support people quarantined at home by providing food. Nine of the ten participants preferred home quarantine over quarantine facilities and described the advantages of home quarantine, including psychological support and low cost.

### Challenges in contact tracing and quarantine

All the KII participants noted that the challenges of dealing with a novel disease with limited information initially about transmission dynamics. According to the participants, political interference, lack of coordination, limited human resources, difficult logistics, and limited epidemiologists in the emergency response team were some of the challenges at the national level in some countries. At the provincial and community level, lack of context-appropriate policies, limited human resources, a lack of training, a lack of incentives and motivation, incomplete information on cases and contacts, delays in receiving laboratory results, difficulties with timely identification of cases, language, lack of community cooperation and trust, limited transport facilities, lack of protective gears, stigma, fear and discrimination, were some of the challenges related to COVID-19 contact tracing and quarantine.

The participants also mentioned that limited training on contact tracing affected policy implementation. Two participants mentioned that the policies or SOPs on contact tracing were developed around six months after the onset of the COVID-19 pandemic. They had to conduct contact tracing without any training or SOPs. Once the SOPs were developed, a few at the national level received their training online so that they could train others at the sub-national level. One participant said, “*Training was done after five-six months of the onset of COVID-19*. *In the initial days*, *no one knew what to do*. *In the human resources side*, *in the control room also*, *the people managing the contact tracing control room*, *they did not have much experience*. *People from different departments joined the contact tracing team*. *They did not know how to handle the cases of people who had caste issues*, *who had fears*. *No one was trained*.*” -*A Contact Tracing Coordination Officer

Non-cooperation from the community affected the contact tracing. In some instances, the contacts often did not show up when the team visited the house, often closed the doors, or sent dogs around that attacked the contact tracing and quarantine team. Regarding community cooperation, one participant said, “*One of the challenges we faced regarding contact tracing was that the people gave us wrong names*, *addresses*, *or locations*. *They used to provide a general location between several villages*. *When we visited the community and asked around to find the contacts*, *villagers said they did not know anyone with that name*.*”* A Contact Tracing Officer

In the initial stages of the pandemic, most cases were imported, and language was a barrier to communicating with foreign nationals. Some countries did not have a system to record passengers’ information. One of the participants shared, “*Most of the cases were imported…*. *We had difficulties tracing them as we needed a system to record all passengers’ addresses and phone numbers*. *Many foreigners travelled in the same plane and then went to different provinces*. *We had to contact the embassy*, *tourist agencies and local authorities to find these people and let them know they were on a flight with a COVID-19 Case*.*”* A Data Manager

Outbreaks occurred in schools and congregated settings and there was a lack of clear guidance on how to quarantine school children and prisoners. A key informant described the challenges this way- *“There were outbreaks in schools*, *prisons*, *and other congregated settings that host many people*. *There were so many prisoners within a limited space*. *We cannot take these prisoners outside to quarantine in facilities*.*…*. *The whole prison was used as a quarantine facility*. *Due to restrictions in prison*, *we could not implement public health measures easily*.*”* A Surveillance Officer

Many participants reported stigma, fear and discrimination associated with COVID-19 disease and were directed to the contact tracing team and community members, which manifested in many ways. When there was a case in a house, the community started rejecting all the members in that household. Two participants shared that many people died during the Delta outbreak, which triggered fear of death among the team members, who were terrified to visit patients, and do case investigation and contact tracing. The participants also mentioned that, even in hospitals, the team members feared conducting in-person case investigations to identify potential contacts. People stopped testing themselves to avoid COVID-19-related social stigma and being ostracised by community members. The healthcare workers working in quarantine facilities were stigmatised and faced discrimination in the community. The broader community rejected health workers as they perceived them as bringing COVID-19 from hospitals to the community. There was also fear among refugees and ethnic minorities that the government would sterilise the people through food or another mechanism if they went to the quarantine facilities.

Lack of coordination between public and private hospitals and laboratories during the initial days of the pandemic affected the contact tracing. Two participants mentioned that there was a limited number of public health specialists and epidemiologists in the MoH, and communication and leadership issues made it difficult to take effective public health measures during the COVID-19 surge.

One participant said, “*We didn’t have a clear leadership from the beginning*. *We had a conflict of interest between the national and provincial teams*. *When my team leader communicated with the provincial lead*, *it worked well*. *But it did not work with others*. *It was often difficult to communicate with the community health workers because they did not know who we were*. *I had to explain again and again who we were*.*”* -A Rapid Response Team Member.

Eight participants mentioned that data entry, management and timely data analysis affected policy implementation. Seven participants said they did not have a database and entered data in hard copy forms, spreadsheets, or Google sheets. One participant stated- *“So much valuable information was still there*. *They were in a paper-based format or soft copies but were not linked to each other*. *Integration was not there*. *We knew the USA’s and UK’s transmission dynamics because the CDC and UK health authority did that*. *But we did not know the transmission dynamics in our settings due to poor data management and a lack of analysis*.*”-*A Surveillance Officer.

Seven participants mentioned policy gaps by highlighting the imprecise definition of contacts, absence of role clarification of tracers and the lack of guidance on how to quarantine people in congregated settings, breastfeeding mothers, infection prevention and control in quarantine facilities and in high-community transmission areas. In contrast, two participants said that they regularly adapted the policies for the local context and did not see any gaps in the guidelines. One of the participants also shared that people involved in contact tracing were not invited to assist with guideline development, and thus field challenges needed to be reflected in the guidelines.

One participant said, “*the people involved in contact tracing and quarantine guidelines development should have lived experience of contact tracing… how the provinces were going*, *and what challenges they faced*. *In my country*, *people involved in guideline development never conducted contact tracing*. *After drafting the guidelines*, *the MoH organised a workshop and invited leaders from other provinces to comment on the document*. *Again*, *these leaders did not have contact tracing experience and were unaware of field challenges*.*”-A Rapid Response Team member*.

### Importance of risk communication and community engagement in contact tracing and quarantine

Three participants added that risk communication would reduce the stigma associated with COVID infection, and community engagement would improve contact tracing and quarantine. As described by one participant, otherwise, “*When the community does not accept what we’re trying to do*, *the compliance will be minimum*.” A Surveillance Officer

They also highlighted the importance of social and print media and daily briefings on COVID-19 on television to engage the public. Two participants mentioned that local NGOs, faith-based organisations, and local panchayet (committee) could be involved in contact tracing as they knew the context and had good access to the community. Four participants shared mixed experiences of engaging law enforcement agencies in contact tracing. One participant said that police were engaged in contact tracing in their province. They added that the police were not medical personnel trained for contact tracing. Involving the police in the team was not accepted well by the community. They said, “It *was done by the police*, *who were not medical groups*. *They were implementing strict laws*. *They were identifying people like the cases or contacts who did something wrong*. *Community members also felt very bad when police visited their homes and asked for quarantine*.*”-*A Training and Telemedicine Officer.

## Discussion

To the best of our knowledge, this is the first study employing a mixed-methods approach to examine the implementation of COVID-19 contact tracing and quarantine policies in the Indo-Pacific region. The study revealed substantial gaps between global guidance vs national and sub-national policies and local contexts, significantly influencing operationalisation at national, provincial, and district levels across the studied countries. These gaps encompassed limited understanding of COVID-19 transmission dynamics, inadequate leadership and coordination, insufficient training for contact tracing, poor comprehension of guideline recommendations, resource constraints, community resistance, and societal stigmas based on religion, ethnicity, and health conditions. These findings correspond with a 2020 rapid review, identifying insufficient training, coordination issues, mistrust, and stigmatisation as barriers to COVID-19 contact tracing [23].

Between April and July 2022 when the survey was conducted, 14 of the 16 countries that the respondents worked in had both contact tracing and quarantine guidelines, demonstrating the willingness and motivation to use public health tools for control of the pandemic. However, due to evolving transmission dynamics and multiple variants, several member states encountered challenges in contextualising and adapting policy recommendations. Insufficient country-level data analysis and limited involvement of field-level officers in guiding policy development led to a partial reflection of the local context in national policies, widening policy gaps. These gaps included poor understanding of the policy recommendations among field teams due to a lack of formal training; varied public health infrastructures and COVID-19 burdens across countries and regions, scarcity of resources with no dedicated staffing or funding for contact tracing, unclear role delineation for public health workers, hierarchical challenges between national and local team members, and the absence of an integrated data system for timely analysis of transmission dynamics, morbidity, mortality, and high-risk groups. Conversely, countries successfully adopting policies in their local contexts experienced fewer implementation challenges.

WHO recommended prioritising those at the highest risk of COVID-19 impacts and ensuring equity in contact tracing recommendations [24]. Published literature showed that people with comorbidities, older adults, low-income groups and ethnic minorities were disproportionately affected by COVID-19 [25, 26]. Limited integrated databases and data analyses hindered timely identification of vulnerable groups, differing significantly from those in high-income countries. Yet, due to insufficient local data, many countries relied on mathematical models from high-income countries for forecasting, prioritising contact tracing strategies, and making quarantine decisions [27].

The presence of mistrust, social and self-stigma, fear, and discrimination against ethnic minorities suggests that the risk communication and community engagement strategies were inadequate in addressing and preventing COVID-19 related stigma and discrimination in some countries. WHO recommended embedding risk communication and community engagement (RCCE) into contact tracing efforts [28]. Due to limited RCCE activities in contact tracing initiatives, mistrust arose between community members and contact tracing teams, resulting in fear, discrimination, and reluctance to undergo tracing and quarantine. Our study underscores that engaging local NGOs, faith-based organisations, and local administrations reinforce community connections and strengthened contact tracing, and these findings are consistent with prior literature [29]. Future policies should consider engaging these entities in contact tracing initiatives at the outset.

Our study found that participants from high-income countries had more experience and formal training in contact tracing. Limited training in LMICs led to unclear understanding of policy recommendations, particularly regarding quarantine guidelines for specific groups such as pregnant or breastfeeding mothers and caste-based distinctions. Lack of standardised training resulted in varied definitions of contacts and oversight of exposure duration in contact identification. Additionally, unclear role delineation within policy documents complicated coordination among ministries and multi-level officers, causing delays in contact tracing.

Our study’s strengths include participants from 16 countries providing diverse COVID-19 contact tracing experiences. We engaged participants at national, regional, and local levels, including epidemiologists, public health officers, physicians, nurses, FETP fellows, surveillance officers, and data managers, ensuring robust and credible data. The utilisation of mixed-method approaches involving a subset of survey respondents and KII participants reinforced the internal validity of our data. Moreover, cross-checking with selected participants ensured the thorough inclusion of their responses, enhancing the interpretative rigor of our research.

Our study had several limitations. First, our study might be prone to recruitment bias due to unknown source population and convenience sampling, limiting the sample’s representativeness. To mitigate this bias, we took several measures. We clearly defined the study population that our research aimed to represent. Additionally, we diversified our sampling approach by leveraging various sources, including networks of study investigators, social media platforms, and esteemed operational partners like TEPHINET. This strategy aimed to broaden the diversity of our sample. Furthermore, to minimise reporting bias, we employed multiple data collection tools, such as online surveys and key-informant interviews. These methods allowed us to verify the information gathered, ensuring a more comprehensive understanding of the subject matter. Second, as the study was conducted during the COVID-19 pandemic, many public health professionals were involved in emergency responses, limiting their participation or response completion. Therefore, the high non-response rate that we encountered might introduce bias, potentially affecting representativeness and generalisability. Nevertheless, our findings align with prior research on contact tracing and quarantine procedures, suggesting minimal impact from the high non-response rate [10, 14, 30]. Third, exclusion of 42 respondents due to missing information or being outside the Indo-Pacific region might have biased our analyses; however, data collected through KIIs helped address this gap and provided critical information on the barriers. Yet, our defined study population and eligibility criteria in participant recruitment materials and use of multiple sources ensured diverse participation from HICs and LMICs. Despite limitations, our mixed-methods findings can drive improvements in ongoing and future contact tracing efforts, addressing persisting gaps and challenges. Despite our efforts to ensure comprehensiveness, the study had very few or no participants from some of the countries in the Indo-Pacific region. Therefore, interpreting our findings should be approached cautiously, as generalising them to the entire region might be contextually misleading.

Our study recommends context-appropriate and implementable measures for LMICs. We propose a convention of regional stakeholders led by peak international agencies to discuss implementation challenges, experiences, and ideas related to contact tracing and quarantine policies. These discussions can inform policy development at the national level, considering contextual nuances. Annual refresher training on contact tracing and quarantine for public health professionals involved in outbreak prevention and control can be considered. This could especially include behavioral and communication skills, and database integration, to strengthen the workforce. National quarantine guidelines should explicitly address the quarantine of specific groups such as children, pregnant mothers, prisoners and people those experiencing social exclusions, and revised guidelines should consider social, cultural, and infrastructural contexts influencing contact tracing and quarantine. We recommend that local NGOs, faith-based organisations, and local administrations are engaged early on to bolster community connections and enhance the effectiveness of contact tracing efforts. Considering the need for a ‘whole of society response’ during pandemics, similar studies understanding the role of non-health workforce should be considered. Finally, conducting future studies employing a mixed-methods design and incorporating a co-design approach can ensure the development of contextually appropriate and feasible contact tracing and quarantine policies.

Our study examining the first two years of contact tracing and quarantine measures revealed that while most countries adopted global guidance on contact tracing policies, modifications to local contexts were minimal, resulting in implementation gaps. Despite these gaps and challenges, local strategies and community initiatives contributed to success. The findings of this study can aid in revising contact tracing and quarantine policies by addressing gaps and developing strategies to implement culturally acceptable and contextually appropriate contact tracing and quarantine activities during future pandemics.

## Supporting information

S1 Checklist Inclusivity in global research. (DOCX)
